# Real-world management of patients with epidermal growth factor receptor (*EGFR*) mutation-positive non–small-cell lung cancer in the USA

**DOI:** 10.1371/journal.pone.0209709

**Published:** 2019-01-04

**Authors:** Yulin Li, Anita Appius, Thirupathi Pattipaka, Andrea Feyereislova, Adrian Cassidy, Apar Kishor Ganti

**Affiliations:** 1 Pharma Development, F. Hoffmann-La Roche Ltd, Beijing, China; 2 Pharma Development, F. Hoffmann-La Roche Ltd, Basel, Switzerland; 3 VA Nebraska Western Iowa Health Care System, Division of Oncology-Hematology, University of Nebraska Medical Center, Nebraska Medical Center, Omaha, Nebraska, United States of America; Seoul National University College of Pharmacy, REPUBLIC OF KOREA

## Abstract

**Background:**

Randomized phase III trials have established the efficacy of epidermal growth factor receptor (EGFR) tyrosine kinase inhibitors as first-line treatment for *EGFR* mutation-positive advanced non–small-cell lung cancer (*EGFR* Mut+ NSCLC). This retrospective cohort study examined the management patterns and outcomes of patients with *EGFR* Mut+ NSCLC in a real-world setting.

**Materials and methods:**

Data were extracted from the US Flatiron Electronic Health Record-derived database. Adult patients with stage IIIB/IV *EGFR* Mut+ NSCLC (exon 19 deletion or exon 21 L858R mutation) who had received first-line systemic therapy between 2011 and 2016 were included. Demographic and clinical characteristics were analyzed. Outcomes evaluated were time to next treatment (a surrogate for progression-free survival) and overall survival.

**Results:**

Of the 22,258 patients with advanced NSCLC in the database, 961 met the inclusion criteria. Median age was 69.0 years (range: 61–78) and the majority were female (68.0%), with stage IV (93.9%), non-squamous cell carcinoma (97.4%). EGFR tyrosine kinase inhibitors were the most widely prescribed first-line therapy (72.8%). The likelihood of receiving an EGFR tyrosine kinase inhibitor or chemotherapy was unaffected by the type of medical insurance patients had. Patients treated with an EGFR tyrosine kinase inhibitor had significantly longer time to next treatment than those given other first-line systemic therapies (p < 0.0001). There were no significant differences in overall survival according to treatment type.

**Conclusion:**

Results from this large US cohort study reflect those obtained in randomized trials of patients with advanced *EGFR* Mut+ NSCLC and demonstrate their transferability into a real-world setting.

## Introduction

Non–small-cell lung cancer (NSCLC) comprises up to 80% of all newly diagnosed lung cancer cases, and more than half of all patients (57%) are diagnosed with metastatic disease [1]. Despite advances in treatment, 5-year survival rates for advanced NSCLC remain low, at around 15–20% [1–3]. Approximately 19% of Western patients (Europe, North America, or Australia) with NSCLC have tumors that harbor a mutation in the *epidermal growth factor receptor* gene (*EGFR* Mut+) while the corresponding prevalence in Asian patients is around 48% [4]. The introduction of tyrosine kinase inhibitors (TKIs) directed against *EGFR*, such as erlotinib, afatinib, and gefitinib, has improved the outlook for this subgroup of patients [5] and these drugs now represent standard first-line treatment for *EGFR* Mut+ NSCLC [6].

The superiority of EGFR TKIs over platinum-doublet chemotherapy has been demonstrated in a number of randomized, phase III studies in treatment-naïve advanced *EGFR* Mut+ NSCLC [7–16]. For example, erlotinib significantly prolonged progression-free survival (PFS) compared with chemotherapy in the ENSURE study (hazard ratio [HR] 0.34; [95% confidence interval (CI) 0.22–0.51], p < 0.0001; median PFS 11.0 months vs 5.5 months, respectively) [7]. The superiority of gefitinib over chemotherapy was demonstrated in patients with *EGFR* Mut+ NSCLC enrolled in the IPASS study; the HR for progression/death was 0.48 (95% CI 0.36–0.64), p < 0.001; median PFS 10.9 months vs 7.4 months, respectively) [10]. Prolonged PFS relative to chemotherapy was also demonstrated with afatinib in the LUX-Lung 3 trial (HR 0.47 [95% CI 0.34–0.65], p < 0.001; median PFS 13.6 months vs 6.9 months, respectively) [16]. However, overall survival (OS) did not differ significantly between the treatment arms in the ENSURE study (median OS: erlotinib 26.3 months vs chemotherapy 25.5 months) [7], nor in the individual LUX Lung-3 or LUX Lung-6 studies when comparing afatinib with chemotherapy [16].

Despite the wealth of published clinical trial data, information concerning the use of EGFR TKIs in a real-world setting is limited. Real-world data complement results from randomized clinical trials and generate evidence on the effectiveness and use of medical products in daily practice [17], often providing a better understanding of patient response, disease patterns, safety data, and economic outcomes in patients of all ages [18]. This study was designed to evaluate the management and clinical outcomes of patients with *EGFR* Mut+ NSCLC by analyzing real-world data from a large US healthcare database.

## Materials and methods

### Data source

This study used data extracted from the Flatiron Health Cloud-based Oncology Electronic Health Record (EHR)-derived database. The longitudinal database represents one of the most comprehensive sources of real-world evidence in oncology. At the data cut-off date of August 31, 2017, the Flatiron Health database included data from more than 265 cancer clinics (~800 sites of care) representing more than 2 million active US cancer patients available for analysis. This database captures data from both structured and unstructured EHR fields using a technology-enabled abstraction process and multi-pronged quality assurance approaches. Data are refreshed monthly to provide near real-time research-ready datasets [19].

Approval of the study protocol was obtained from Copernicus Group Independent Review Board (CGIRB) prior to study conduct, and included a waiver of informed consent. Data provided to third parties were de-identified and provisions were in place to prevent re-identification in order to protect patients’ confidentiality.

### Patients and methods

This retrospective study examined treatment patterns, demographic and disease characteristics, and clinical outcomes of patients with locally advanced unresectable or metastatic, *EGFR* Mut+ NSCLC. The patient cohort for this study was selected from the Flatiron advanced NSCLC population, which included patients with a diagnosis of lung cancer, ≥2 clinical visits on or after January 1, 2011, and evidence of stage IIIB, IV, or recurrent metastatic NSCLC on or after January 1, 2011.

The selection criteria for this study included: age ≥18 years at diagnosis, histologically confirmed stage IIIB/IV NSCLC (American Joint Committee on Cancer [AJCC], 7th edition), documented *EGFR* Mut+ disease (exon 19 deletion or exon 21 L858R mutation), and receipt of systemic therapy within 90 days of diagnosis and between January 1, 2011–June 30, 2016. Patients with evidence of data discrepancies, such as diagnosis date before birth year, date of treatment initiation before diagnosis date, mortality date before last visit date, were excluded. The start of treatment of interest in the first-line setting was defined as the index date to evaluate clinical outcomes for the treatment subgroup. Patients were retrospectively followed up until June 30, 2017, to allow at least 1-year follow up for each patient.

### Study endpoints

Demographic variables evaluated were age, gender, race, and smoking status. Disease clinical characteristics included tumor histology, stage at diagnosis, absence or presence of brain metastases, Eastern Cooperative Oncology Group performance status, and *EGFR* mutation information. The proportions and sequence of patients receiving chemotherapy and targeted agents, with a focus on TKIs, were examined.

Time to next treatment (TTNT) was used as a surrogate for PFS and was defined as the time from the start of first-line treatment for advanced *EGFR* Mut+ NSCLC until initiation of second-line treatment, discontinuation of first-line treatment, the data cut-off, or death. OS was defined as the time from initiation of first-line treatment until death from any cause. Due to the de-identification processing of the analysis dataset, all death dates were approximated to the 15th of the month. Patients were censored at the last visit date or at the data cut-off date, whichever occurred first, if they had no death information or if they were still on treatment at the end of the study period.

The predefined variable of line of therapy in the Flatiron database was used to define the first-line setting. However, since this variable was derived from administrative or prescription dates, and name and dosage of regimens, rather than date of disease progression, line of therapy described in the study should be interpreted as a surrogate to line of therapy in a clinical trial.

### Statistical analyses

Baseline patient and clinical characteristics were described at the start of the first-line treatment in the overall study cohort and by treatment subgroups. Categorical variables were described as frequency and percentages. For continuous variables, means, standard deviations, medians, range and percentiles (25th and 75th) were reported when appropriate. Comparisons between treatment subgroups were made using chi-square tests for categorical variables and Wilcoxon rank-sum test for continuous variables (two-sided test, p-value threshold ≤ 0.05). No formal hypotheses were tested and p-values were reported purely for descriptive purposes. All analyses were performed in the overall study cohort and by treatment subgroups (e.g., TKIs, erlotinib, afatinib, chemotherapy, or other non-TKI target therapy in the first-line). A Kaplan–Meier analysis was conducted to estimate median TTNT and OS with 95% CIs; HRs and 95% CIs with p-values were calculated using a Cox-proportional hazards model after adjusting for patient age, gender, race, smoking status, and histology. All statistical analyses were conducted using SAS software (SAS Institute, Cary, NC) or R.

## Results

### Patients

**Fig 1:**
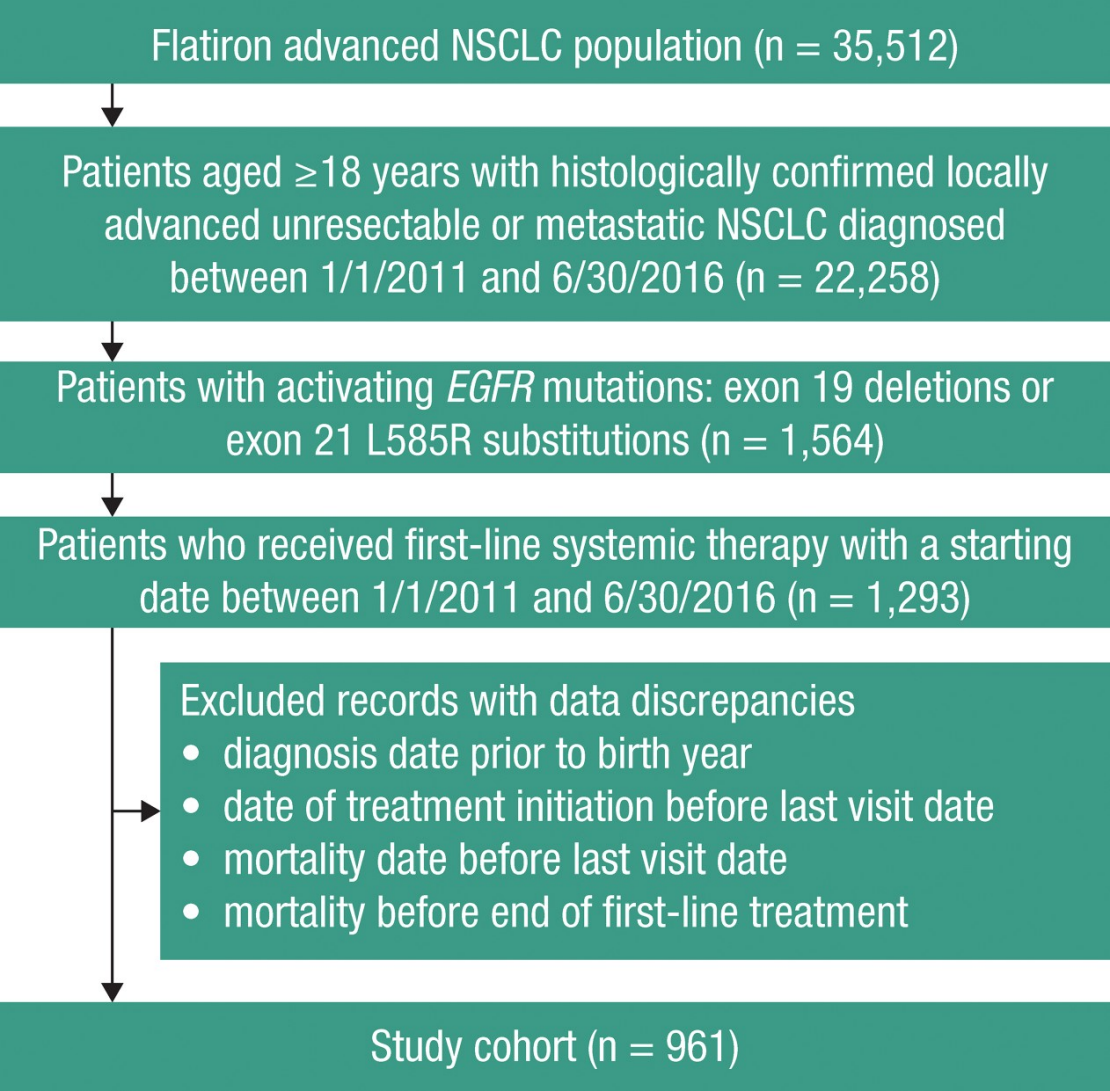
Participant flow through the study.

In total, 22,258 patients aged ≥18 years with stage IIIB/IV NSCLC were identified from the Flatiron advanced NSCLC cohort (Fig 1). Of these, 1,564 patients had an activating *EGFR* mutation in their tumors, and 1,293 patients received first-line systemic therapy for NSCLC between January 1, 2011–June 30, 2016. After excluding patients with evidence of data discrepancies, a total of 961 patients were included in the main analysis. Since only 13 patients were treated with gefitinib, these patients were not included in subgroup analyses.

### Baseline demographic and clinical characteristics

Patients were older than the median age observed in clinical trials of *EGFR* Mut+ NSCLC (~60 years) [7,13,16] with a median age of 69 years (range: 61–78) (Table 1). The majority of patients were female (68.0%, n = 653), with stage IV (93.9%, n = 902) non-squamous non-small cell carcinoma (97.4%, n = 936). Symptomatic brain metastases were absent or not prospectively diagnosed for a large proportion of patients (83.7%, n = 804). Most patients were treated within a community setting (91.3%, n = 877) and received monotherapy (69.0%, n = 663) as first-line treatment. No obvious differences in other demographic or clinical characteristics were observed among the treatment groups.

**Table 1:** Baseline patient characteristics.

	All NSCLC patients (n = 961)		EGFR TKIs (n = 700)		Afatinib (n = 87)		Erlotinib (n = 593)		Gefitinib (n = 13)^a^		Chemotherapy only (n = 169)		Chemotherapy + non-TKI targeted therapy (n = 85)	
	n	%	n	%	n	%	n	%	n	%	n	%	n	%
Age at advanced diagnosis, years														
18–64	341	35.5	236	33.7	24	27.6	207	34.9	1	7.7	62	36.7	40	47.1
65–74	309	32.2	208	29.7	25	28.7	179	30.2	3	23.1	68	40.2	29	34.1
75+	311	32.4	256	36.6	38	43.7	207	34.9	9	69.2	39	23.1	16	18.8
Median (*range*)	69 (61–78)		69 (62–79)		69 (62–81)		69 (62–78)		79 (74–84)		68 (59–74)		65 (60–72)	
Gender														
Female	653	68.0	488	69.7	62	71.3	410	69.1	12	92.3	103	61.0	58	68.2
Male	308	32.1	212	30.3	25	28.7	183	30.9	1	7.7	66	39.1	27	31.8
Race														
Asian	122	12.7	102	14.6	10	11.5	89	15.0	2	15.4	16	19.5	3	3.5
Black / African American	55	5.7	38	5.4	4	4.6	33	5.6	1	7.7	11	6.5	6	7.1
Hispanic or Latino	6	0.6	6	0.9	3	3.5	2	0.3	0	0	0	0	0	0
Other race	117	12.2	89	12.7	12	13.8	73	12.3	4	30.8	14	8.3	14	16.5
White	532	55.4	371	53.0	43	49.4	321	54.1	4	30.8	106	62.7	50	58.8
Missing	129	13.4	94	13.4	15	17.2	75	12.7	2	15.4	22	13.0	12	14.1
Smoking status														
History of smoking	423	44.0	289	41.3	37	42.5	245	41.3	5	38.5	92	54.4	42	49.4
No history of smoking	525	54.6	404	57.7	50	57.5	341	57.5	8	61.5	74	43.8	41	48.2
Unknown/ not documented	13	1.4	7	1.0	0	0	7	1.2	0	0	3	1.8	2	2.4
Histology														
NSCLC histology NOS	12	1.3	4	0.6	0	0	4	0.7	0	0	6	3.6	1	1.2
Non-squamous NSCLC	936	97.4	692	98.9	86	98.9	586	98.8	13	100	154	91.1	84	98.8
Squamous cell carcinoma	13	1.4	4	0.6	1	1.2	3	0.5	0	0	9	5.3	0	0
Clinical stage														
IIIB	59	6.1	2	2.9	2	2.3	18	3.0	0	0	36	21.3	3	3.5
IV	902	93.9	680	97.1	85	97.7	575	97.0	13	100	133	78.7	82	96.5
Brain metastases														
Absent^b^	804	83.7	586	83.7	72	82.8	498	84.0	11	84.6	136	80.5	76	89.4
Diagnosed after treatment	81	8.4	54	7.7	7	8.1	46	7.8	1	7.7	18	10.7	8	9.4
Diagnosed before treatment	76	7.9	60	8.6	8	9.2	49	8.3	1	7.7	15	8.9	1	1.2
ECOG PS (± 90 days of diagnosis)														
0–1	342	35.6	263	37.6	31	35.6	221	37.3	6	46.2	49	29.0	29	34.1
2+	85	8.8	68	9.7	10	11.5	55	9.3	3	23.1	11	6.5	6	7.1
Unknown	534	55.6	369	52.7	46	52.9	317	53.5	4	30.8	109	64.5	50	58.8
Practice type														
Academic	84	8.7	71	10.1	6	6.9	65	11.0	0	0	9	5.3	1	1.2
Community	877	91.3	629	89.9	81	93.1	528	89.0	13	100	160	94.7	84	98.8
Insurance type														
Commercial health plan	367	38.2	258	36.9	29	33.3	221	37.3	3	23.1	69	40.8	35	41.2
Medicaid	29	3.0	24	3.4	2	2.3	22	3.7	0	0	3	1.8	2	2.4
Medicare	145	15.1	109	15.6	21	24.1	84	14.2	4	30.8	23	13.6	12	14.1
Missing	167	17.4	115	16.4	14	16.1	97	16.4	4	30.8	32	18.9	20	23.5
Other government program	24	2.5	8	2.6	1	1.2	17	2.9	0	0	3	1.8	3	3.5
Other payer–type unknown	190	19.8	151	21.6	17	19.5	130	21.9	2	15.4	30	17.8	8	9.4
Patient-assistance program	37	3.9	23	3.3	3	3.5	20	3.4	0	0	9	5.3	5	5.9
Self-pay	2	0.2	2	0.3	0	0	2	0.3	0	0	0	0	0	0

^a^ Data for patients who received gefitinib were removed from all analyses due to the small sample size (n = 13).

^b^ No secondary malignant brain, spinal cord, or nervous system neoplasm present during the study period.

CTX, chemotherapy; ECOG PS, Eastern Cooperative Oncology Group performance status; NSCLC, non-small-cell lung cancer; NOS, not otherwise specified; EGFR TKIs, epidermal growth factor receptor tyrosine kinase inhibitors

### Biomarker testing

**Fig 2:**
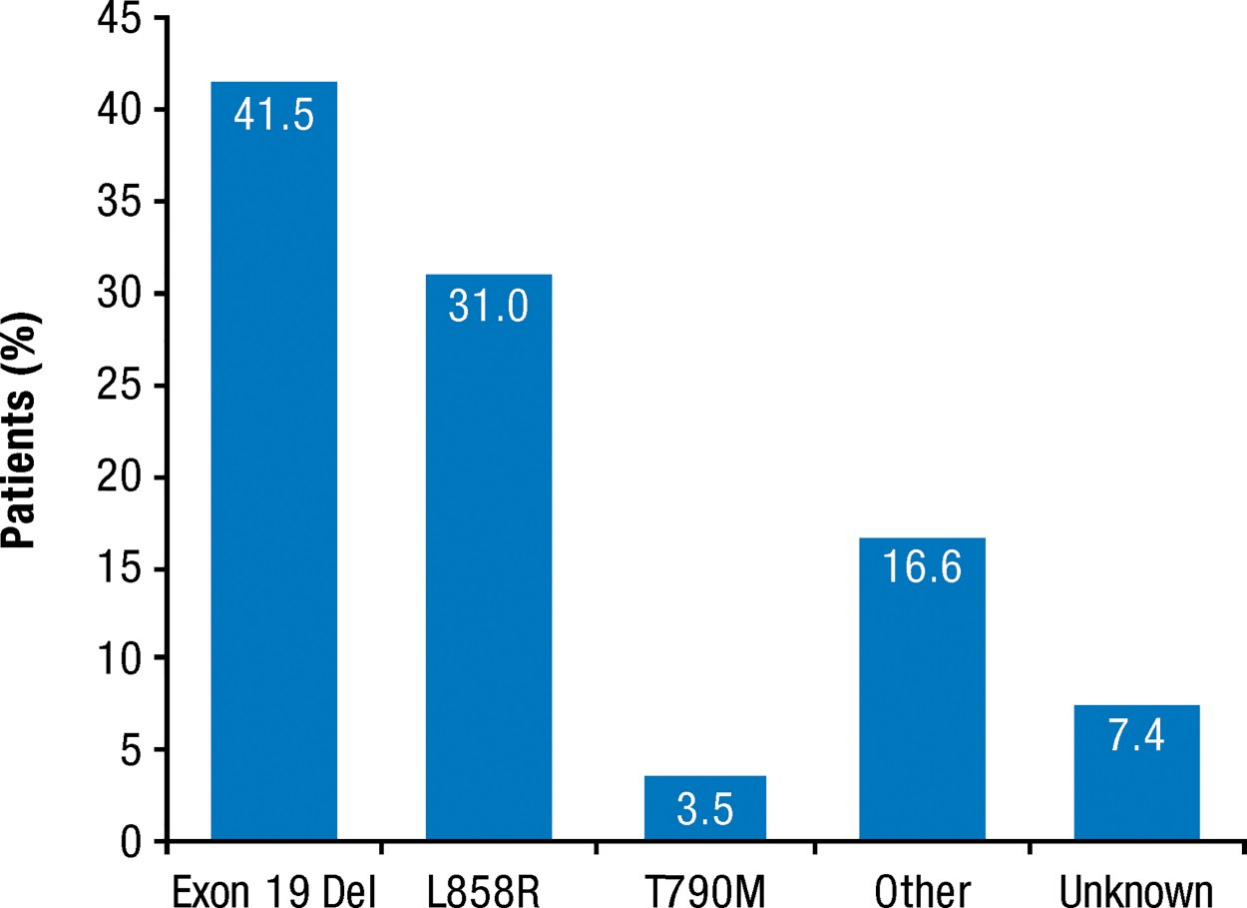
Frequency of the most common *EGFR* mutations (n = 2,080).

Rates of *EGFR* mutation testing steadily rose from 30.5% in 2011 to 78.4% in 2016 (Table 2). Of 12,148 patients who underwent *EGFR* testing, 17.1% (n = 2,080) had a confirmed diagnosis of *EGFR* Mut+ NSCLC, with exon 19 deletion (41.5%, n = 864) or exon 21 L858R point mutation (31.0%, n = 645) identified most frequently (Fig 2). *De novo* T790 mutations were present in 3.5% of patients (n = 73) and other/unknown *EGFR* mutations in 24.0% of patients (n = 499).

**Table 2:** *EGFR* mutation testing rates by year in 12,148 patients who underwent *EGFR* testing.

Year	Patients diagnosed^a^	Testing rate
	n	%
2010	–	–
2011	2,650	30.5
2012	3,420	48.0
2013	4,093	55.0
2014	4,599	60.5
2015	4,934	63.9
2016^b^	2,562	78.4

^a^ Patients may be tested in different years to their year of diagnosis.

^b^ Testing was only undertaken for a 6-month period up to June 30, 2016.

Note: The patient cohort presented in this table is larger than the cohort described in the methods.

### Treatment patterns

Of the 961 patients in the main analysis, EGFR TKIs (72.8%, n = 700) were the most widely prescribed first-line therapy (erlotinib 61.7% [n = 593], afatinib 9.1% [n = 87], gefitinib 1.4% [n = 13]). Seven patients received a combination of these TKIs. First-line chemotherapy was prescribed to 17.6% (n = 169) of patients and first-line chemotherapy ± non-EGFR TKI targeted therapy (either bevacizumab or cetuximab) to 8.8% of patients (n = 85) (Table 3).

**Table 3:** Frequency of first- and second-line treatment.

First-line regimen, n		Second-line regimen, n							
	None	Erlotinib	Afatinib	Gefitinib	TKI combination	Chemotherapy	Targeted drug	Clinical study drug	Total, n (%^d^)
Erlotinib	365	23	57	9	4	91	38	6	593 (61.7)
Afatinib	67	7	1	0	1	4	7	0	87 (9.1)
Gefitinib	13	0	0	0	0	0	0	0	13 (1.4)
TKI combination^a^	6	0	0	0	0	1	0	0	7 (0.7)
Chemotherapy^b^	54	74	9	1	0	28	3	0	169 (17.6)
Targeted drug^c^	29	32	1	1	0	13	9	0	85 (8.8)
Clinical study drug	5	2	0	0	0	0	0	0	7 (0.7)
Total, *n* (%^e^)	539	138 (32.7)	68 (16.1)	11 (2.6)	5 (1.2)	137 (32.5)	57 (0.1)	6 (1.4)	961

^a^ Patients who received a combination of erlotinib or afatinib or gefitinib in the examined line.

^b^ Patients who received chemotherapy without combination use of any EGFR TKI or non-EGFR TKI targeted treatments.

^c^ Patients who received at least one non-EGFR TKI targeted treatment in the examined line.

^d^ Denominator = 961 patients who received first-line treatment.

^e^ Denominator = 442 patients who received second-line treatment.

TKI, tyrosine kinase inhibitor

Amongst patients receiving treatments other than EGFR TKIs (n = 261), carboplatin/pemetrexed (23.4%, n = 61), carboplatin/paclitaxel (20.3%, n = 53), and bevacizumab/carboplatin/pemetrexed (16.9%, n = 44) were the most frequently prescribed first-line therapies.

Fewer than half of all patients (43.9%, n = 422) received second-line treatment (Table 3), which was most commonly erlotinib (32.7%, n = 138), chemotherapy (32.5%, n = 137), or afatinib (16.1%, n = 68).

### Insurance

The likelihood of receiving an EGFR TKI was generally unaffected by the type of medical insurance patients had (Table 1). The most common insurance type was a commercial health plan, which was held by 36.9% (n = 258) of patients who received EGFR TKIs, 40.8% (n = 99) of patients who received chemotherapy alone and by 41.2% (n = 35) of patients on other non-TKI targeted treatments (Table 1). Patterns of insurance were similar among patients receiving an EGFR TKI and patients receiving other systemic therapy.

### Clinical outcomes

**Fig 3:**
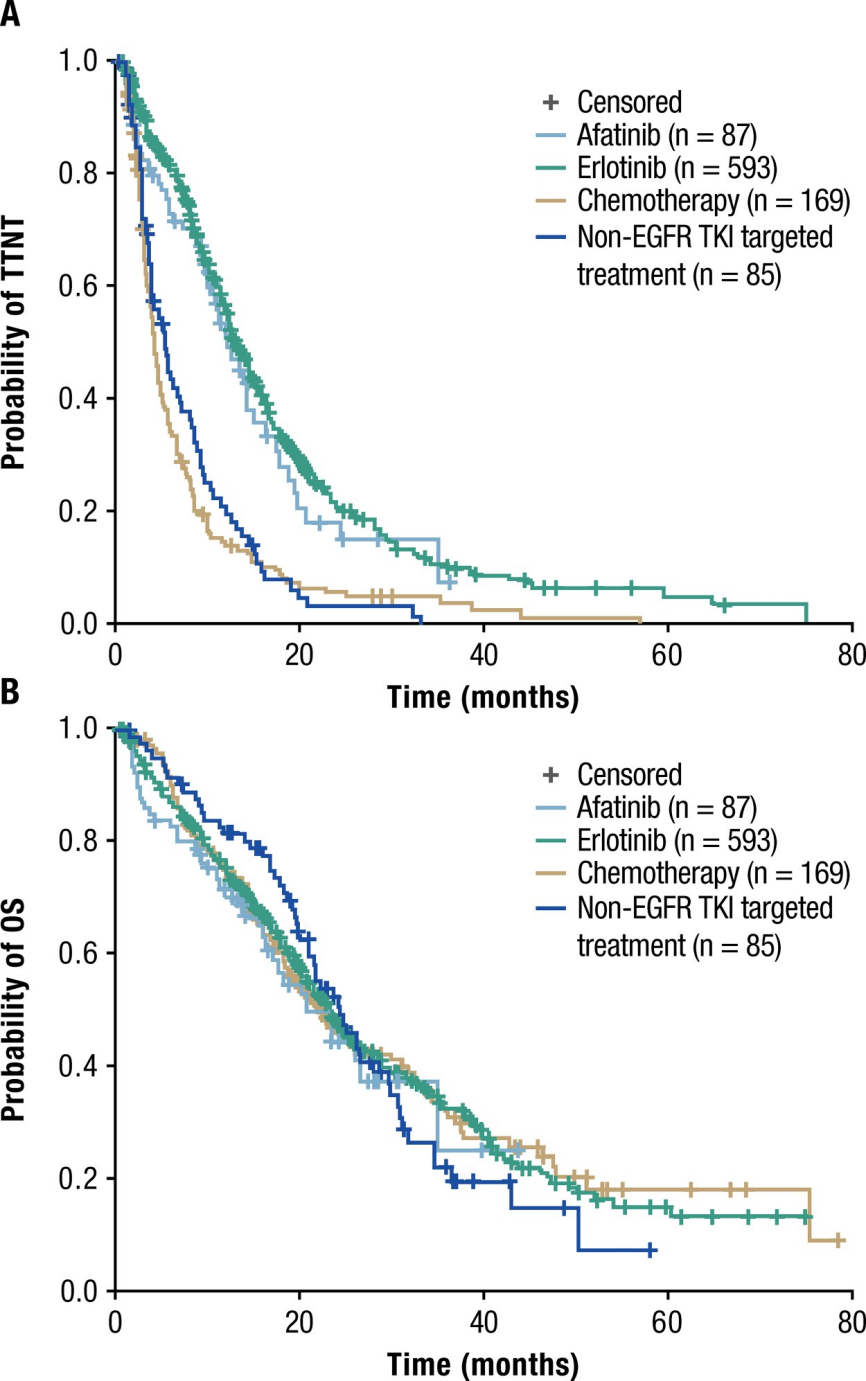
Kaplan-Meier estimates of (A) TTNT, and (B) OS stratified by treatment group.

Patients who received erlotinib (n = 593) or afatinib (n = 87) as their first-line therapy had a significantly longer TTNT than patients receiving non-EGFR TKI therapy (n = 261) (both p < 0.0001; Fig 3A). Median TTNT was 13.1 months (95% CI 12.1–14.3) in the erlotinib group, 12.1 months (95% CI 9.7–14.9) in the afatinib group, 5.3 months (95% CI 3.7–7.2) in the non-EGFR TKI targeted treatment group, and 4.2 months (95% CI 3.7–4.9) for patients receiving chemotherapy.

Among the 961 patients included in the main analysis, 549 patients (57.1%) had died at the time of the analysis. These included 40 (46% of 87), 338 (57% of 593), and 104 (61.5% of 169) patients treated with afatinib, erlotinib, and chemotherapy, respectively. Estimated median OS with erlotinib (median 23.2 months, 95% CI 21.2–24.9) and afatinib (median 20.7 months, 95% CI 16.2–35.1) was comparable to that seen with non-EGFR TKI targeted treatment (median 24.1 months, 95% CI 20.9–29.5) and chemotherapy (median 22.1 months, 95% CI 18.3–30.0) (Fig 3B).

**Fig 4:**
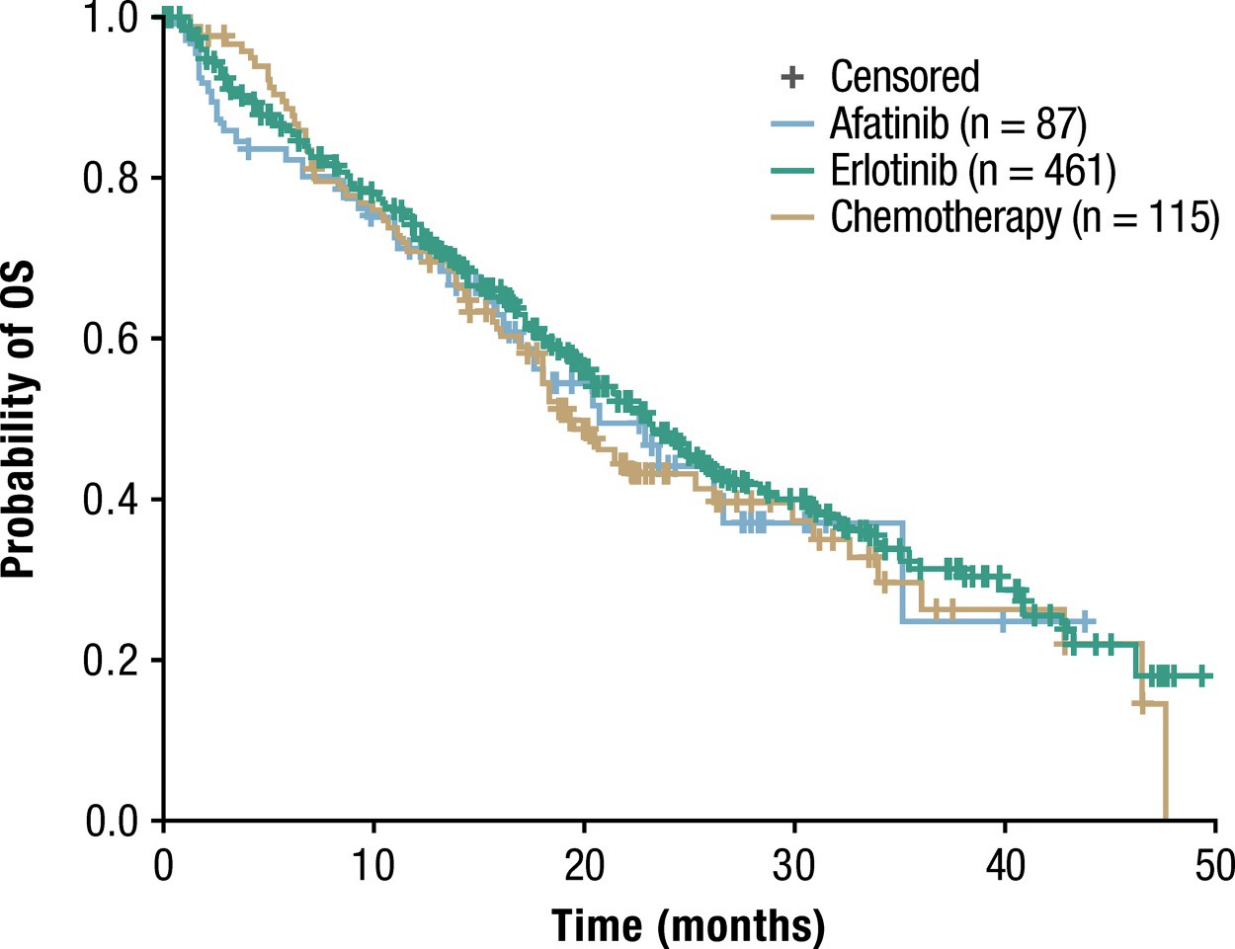
Kaplan-Meier estimates of OS stratified by treatment group in patients diagnosed after May 2013.

Similar results were seen in exploratory subgroup analyses in patients diagnosed with advanced *EGFR* Mut+ NSCLC after May 2013 (n = 744), following the US Food and Drug Administration approval of afatinib in this setting. Median OS with erlotinib (n = 461) was 23.1 months (95% CI 20.4–25.5), compared with 20.7 months (95% CI 16.2–35.1) with afatinib (n = 87) and 19.3 months (95% CI 17.0–26.2) with chemotherapy (n = 115) (Fig 4).

Exon 19 deletion was identified in 52 patients in the afatinib group and 326 patients in the erlotinib group, and exon 21 mutation was found in 35 and 268 patients, respectively. Median TTNT was similar between these treatment groups for both mutations; median TTNT was 12.6 months (95% CI 9.0–17.5) with afatinib and 14.0 months (95% CI 12.4–15.3) with erlotinib for patients with an exon 19 deletion, and 11.2 months (95% CI 8.7–16.2) and 12.1 months (95% CI 10.6–14.2), respectively, for those with an exon 21 mutation (S1 Table). Median OS appeared numerically longer in patients whose tumors harbored an exon 21 mutation treated with erlotinib (19.9 months, 95% CI 17.3–24.2) vs afatinib (16.2 months, 95% CI 11.0–26.1), but this difference was not statistically significant. In patients with exon 19 deletion, median OS was similar between the treatment groups: 23.0 months (95% CI 18.1–not reached) with afatinib and 24.6 months (95% CI 23.2–29.0) with erlotinib.

## Discussion

This retrospective study evaluated real-world treatment patterns of patients with advanced *EGFR* Mut+ NSCLC in oncology practices across the USA, and examined demographic and disease characteristics among patients receiving EGFR TKIs vs other systemic anti-cancer therapies. With the exception of age (clinical trial patients are approximately 9 years younger), the baseline characteristics of the patients eligible for this analysis were similar to those observed in randomized, clinical trials of *EGFR* Mut+ NSCLC [7,13,16].

The rate of *EGFR* mutation testing in our analysis was low in 2011, at just 30.5%. This contrasts with rates of up to 65% reported in Japan during the same year, although testing rates in China were only 18% [20]. Following a steady increase in testing rates between 2011 and 2015, we observed a rise in *EGFR* mutation testing during 2016 to approximately 78%. Apart from the potential bias introduced by data completeness during the earlier years, this increase probably reflects an enhanced awareness of the need for *EGFR* mutation testing in patients with advanced NSCLC before initiation of first-line therapy, as recommended in clinical practice guidelines published at that time [21,22], or to a wider availability of a validated test. Notably, the results of a retrospective chart review in patients with advanced non-squamous NSCLC initiating therapy between 2011 and 2013, with follow-up until 2016, revealed *EGFR* mutation testing rates of more than 97% in Taiwan [23]. Compared with our study population, fewer of the Taiwanese patients were aged >75 years (22% vs 32%, respectively), with a history of smoking (33% vs 44%, respectively), and a greater proportion of them received first-line therapy for advanced NSCLC (100% vs 83%, respectively) [23]. Reports from the Western literature during the same time-period have suggested testing rates in the region of 70%, congruent with the results of our analysis [24]. These results suggest that the introduction of the broad use of molecular testing took more than 5 years to achieve levels of almost 80%, assuming that in about 5–10% of patients tumor testing cannot be performed for various reasons (e.g., no tumor tissue available, no biopsy possible, or need for immediate treatment intervention without a delay).

Treatment patterns were as expected, based on the mutation status of the patients’ tumors, with single-agent EGFR TKIs, predominantly erlotinib, having been the most widely prescribed first-line therapy. The type of medical insurance that patients had did not influence the likelihood of them receiving an EGFR TKI. There has been a lack of evidence supporting the efficacy of combining EGFR TKIs with chemotherapy, nevertheless, erlotinib and afatinib were used in combination with chemotherapy in a few patients (n = 32; 3.3% of the entire cohort). We speculate that a TKI was intercalated with chemotherapy rather than given concomitantly.

Approximately 27% of patients with *EGFR* Mut+ NSCLC were treated with a platinum-containing chemotherapy doublet either with or without bevacizumab. Single-agent erlotinib was the second-line therapy of choice in the majority of patients who continued with treatment, presumably given to those patients who did not receive a TKI as first-line treatment.

Patients who were given an EGFR TKI as first-line therapy had significantly longer TTNT, a surrogate for PFS, than those receiving other systemic treatments. Median TTNT was 13.1 months (95% CI 12.1–14.3) with erlotinib and 12.1 months (95% CI 9.7–14.9) with afatinib. Erlotinib and afatinib demonstrated comparable efficacy in this regard (both p < 0.0001 vs other systemic therapies). The slightly longer duration of TTNT in this study compared with PFS in clinical trials [7–9, 13–15] most likely reflects the time that would typically elapse between clinical or radiologic progression and the start of the next course of treatment in eligible patients. The comparable values seen for TTNT in this real-world setting and PFS observed in a clinical trial setting are important and reassuring. The patients in our study had a higher median age than patients enrolled in clinical trials, confirming that increased age is not associated with reduced efficacy in the real-world setting. As observed in randomized studies [7,25], no significant difference in OS was noted among patients receiving EGFR TKIs vs those receiving chemotherapy as their initial therapy. This may be because almost half of the patients who received chemotherapy initially, received an EGFR TKI on progression (49.7%, n = 84/169). However, 31.9% of patients (n = 54) who received chemotherapy upfront were never exposed to an EGFR TKI. While the reason for this may vary, this finding requires further investigation. The median OS of 23.2 months (95% CI 21.2–24.9) achieved with erlotinib and 20.7 months (95% CI 16.2–35.1) achieved with afatinib were in-line with the survival times reported in phase III studies [7,11,16]. Of note, a much higher number of patients received treatment with erlotinib than with afatinib throughout the study (n = 593 vs n = 87, respectively).

The results from this real-world study support the efficacy of the EGFR TKIs established in clinical trials. However, the population studied in the USA may not be representative of a global population, and additional analyses may be needed to evaluate real-world treatment patterns in other countries. In a retrospective chart review examining real-world practice patterns in 175 Japanese patients treated for stage IIB/IV NSCLC between 2011 and 2013, EGFR TKIs were the most commonly prescribed therapies for *EGFR* Mut+ NSCLC across all treatment lines [26], similar to our findings. Of note, median OS from the start of first-line therapy was 9.9 months (95% CI 7.6–11.7) for all patients compared with 17.9 months (95% CI 9.9–24.4) for those with *EGFR*/*ALK* Mut+ tumors. The authors note that due to the retrospective nature of the study, these results are not reflective of the current clinical landscape, given the availability of newer therapies for NSCLC, namely third-generation EGFR TKIs [26,27].

Limitations of this analysis are reflective of the data source and collection. Flatiron data are generated from real-world clinical practice, and are subject to miscoding and errors. The data are mainly drawn from community oncology centers and therefore may not be representative of treatment patterns at academic medical centers. Since information about patients prior to the clinical oncology practice’s adoption of the EHR may not be available, particularly in the structured data, the extent to which historical data are entered into the EHR is a practice-specific decision and varies widely. An important strength of the present study over previous real-world data studies [28,29] was the availability of information on mortality in patients with *EGFR* Mut+ NSCLC from which OS data could be estimated. Moreover, the sensitivity and specificity of mortality in the advanced NSCLC dataset is 91% and 96%, respectively, which supports its application to evaluate clinical outcomes in this setting [30]. Finally, the study enabled a description of longitudinal treatment patterns with a minimum of 12 months of follow-up for every patient. Notwithstanding the limitations, our results represent the largest available real-world data in this setting. Concurrence between these data and clinical trial evidence is reassuring, with respect to the accuracy and validity of our findings on one hand and applicability of the clinical data in real-life practice on the other.

## Conclusions

These real-world data reflect the results of randomized clinical trials of *EGFR* Mut+ NSCLC, with the exception of patients in this analysis being generally older, by approximately 9 years. Testing rates for *EGFR* mutations steadily increased over time, reaching approximately 78% in 2016, although there is room for improvement. The majority of patients with *EGFR* Mut+ disease received EGFR TKIs as first-line therapy, which were predominantly administered as monotherapy. Patients who received first-line EGFR TKIs had significantly longer TTNT than those who received other non-EGFR TKI systemic therapies. No significant differences in OS were observed by treatment type.

## Supporting information

S1 Table TTNT and OS outcomes by *EGFR* mutation status. (DOCX)
