# Correction: Real-world management of patients with epidermal growth factor receptor (*EGFR*) mutation-positive non-small-cell lung cancer in the USA

**DOI:** 10.1371/journal.pone.0212831

**Published:** 2019-02-20

**Authors:** Yulin Li, Anita Appius, Thirupathi Pattipaka, Andrea Feyereislova, Adrian Cassidy, Apar Kishor Ganti

The first author, Yulin Li, is incorrectly noted as the corresponding author. The correct corresponding author is Apar Kishor Ganti. Dr. Ganti's email address is: aganti@unmc.edu.
