# Supplementary material for: Real-world management of patients with epidermal growth factor receptor (EGFR) mutation-positive non–small-cell lung cancer in the USA
Source: PLoS One. 2019 Jan 4;14(1):e0209709. doi: 10.1371/journal.pone.0209709 (PMC6319739; doi:10.1371/journal.pone.0209709)
Supplement: S1 Table — (DOCX) [file pone.0209709.s001.docx]

**Supporting Information**

**S1 Table. TTNT and OS outcomes by *EGFR* mutation status.**

|  | **Exon 19 deletion** | | | **Exon 21 mutation** | | |
| --- | --- | --- | --- | --- | --- | --- |
|  | **Afatinib  (n = 52)** | **Erlotinib  (n = 325)** | **p** | **Afatinib  (n = 35)** | **Erlotinib**  **(n = 268)** | **p** |
| Median TTNT, months (95% CI) | 12.6  (9.0–17.5) | 14.0  (12.4–15.3) | 0.33 | 11.2  (8.7–16.2) | 12.1  (10.6–14.2) | 0.63 |
| Median OS, months  (95% CI) | 23.0  (18.1–not reached) | 24.6  (23.2–29.0) | 0.89 | 16.2  (11.0–26.1) | 19.9  (17.3–24.2) | 0.18 |

EGFR, epidermal growth factor receptor; OS, overall survival; TTNT, time to next treatment.
